# Strand-Morphology-Based Process Optimization for Extrusion-Based Silicone Additive Manufacturing

**DOI:** 10.3390/polym13203576

**Published:** 2021-10-16

**Authors:** Dingyifei Ma, Xiaoqing Tian, Shengyi Wang, Haijun Liu, Shan Chen, Jiang Han, Lian Xia

**Affiliations:** 1 School of Mechanical Engineering, Hefei University of Technology, Hefei 230009, China; 2018110072@mail.hfut.edu.cn (D.M.); shey93@126.com (S.W.); liuhaijun@hfut.edu.cn (H.L.); cs0305@hfut.edu.cn (S.C.); xialian@hfut.edu.cn (L.X.); 2 Anhui Engineering Laboratory of Intelligent CNC Technology and Equipment, Hefei 230009, China

**Keywords:** material extrusion, tensile test, silicone, additive manufacturing

## Abstract

In the silicone material extrusion (MEX) process, product profile error and performance defects are common problems due to changes in strand shape. A process optimization method considering strand morphology, denoted as SMO, which allows adjustment of the strand shape by adjusting process parameters during the printing process is presented. The relation between process parameters (extrusion speed, moving speed, nozzle height, and nozzle radius) and the geometric parameters (strand width and strand height) of the cross-section, as well as the relationship between strand spacing, layer height, and process parameters in no void constraint is discussed and verified. SMO was utilized to produce specimens with tunable strand width and strand height. Tensile tests and profile scans were performed to compare SMO with other methods to verify its feasibility. Specimens fabricated using the SMO method have up to a 7% increase in tensile strength, up to a 10% reduction in processing time, and about a 60% reduction in strand height error over unused ones. The results show that the SMO method with adjustable strand width can effectively balance efficiency and mechanical properties compared to uniform infill, and the SMO method with adjustable strand height can provide higher accuracy compared to uniform strand height. The proposed method is validated and improves the efficiency and accuracy of silicone MEX.

## 1. Introduction

The material extrusion (MEX) method [1] for silicone is used in scaffolds for tissue engineering [2], stretchable electronics [3], manufacturing of soft robots [3], orthoses and prostheses [4], drug-delivery devices, nonwovens [5], and other components with flexible thin-walled structure, complex internal structure, as well as high elongation and fatigue life, as silicone material is an environmentally friendly raw material, with superior characteristics of softness and non-toxicity. This process demonstrates better silicone printing capability than other processes due to its compatibility with a wide range of commercially available silicone materials, with tensile strength hardness of 3 to 90 Shore A, elongation of 500% to 1200% [2], functional temperature of −65 to 177 °C, and chemical resistance. Compared to traditional methods, additive manufacturing (AM) is cost effective [6] as it can effectively reduce the cost of AM products and improve productivity through customization, rapid prototyping, and geometric freedom by using different optimization methods in the design, including topology optimization [7], support optimization and selection of part orientation [8,9], and part consolidation [10]. Silicone additive manufacturing has advantages over traditional manufacturing (such as molding and soft lithography) in terms of complicated geometry and undercut feature fabrication, production cost, and production cycle time [1].

After extrusion-based silicone additive manufacturing (AM) was first applied to prepare silicone parts in 2014 [11], the reactive-based inkjet printing method [12] (material jetting two-part curing), three-dimension direct-writing thermo-curing (3DDT) technology [13,14] (MEX thermal curing), drop-on-demand technology [15] (material jetting UV curing), extrusion-based moisture curing technology [16], etc., were developed. Moisture curing methods were combined with the liquid rope coiling effect to fabricate silicone foam with variable elastic modulus [17] and three-dimensional contour nonwoven fabrics [5] to exceed the limits of silicone MEX [18]; A theoretical model for silicone MEX was established and verified to effectively control the speed and accuracy [3]. 

The printing quality control of silicone MEX is one of the key issues. The optimization of the silicone MEX process is mainly carried out in terms of equipment structure optimization [18,19]; new materials; process parameter optimization [8,19,20,21]; process research considering material properties [22], rheological properties [23], forces [24], or other factors; slicing [25]; path planning [26]; motion control [27]; etc. 

The silicone MEX process can be optimized by controlling the strand width. Comminal et al. [28,29] studied the MEX process by means of computational fluid dynamics (CFD). The influence of process parameters on the shape of the strand section was analyzed, as well as the extrusion process of semi-molten materials at the corners, and the print quality was improved through synchronization of extrusion and moving speed. Liravi et al. [30] developed a hybrid AM method that combines MEX and material jetting technologies for silicone-based bio-structure fabrication. Muthusamy et al. [31] developed a process achieving silicone parts with overhang structure fabrication, using poly-vinyl alcohol (PVA) as supporting material, which extends the processing range of silicone extrusion additive manufacturing. Miao et al. [32] studied the effect of printing process parameters on the printing quality of 3D printed metal blanks. They studied the synchronization of the flow rate and moving speed during printing and obtained the theoretical formula for the flow rate and print speed synchronization. They also studied the proportional relationship between the layering height and the nozzle diameter. Results showed that when the layering height is 70% to 80% of the nozzle diameter, the molding quality is good. Chi et al. [33] explored the effect of 3D printing process parameters on product performance. They researched orthogonal experiments and analyzed the influence of process parameters on the tensile strength and elongation at the break of the specimen. The degree of influence is plasticizing temperature >layer height > filling angle. Foteinopoulos et al. [34] studied the factors (extrusion speed, moving speed) affecting the quality of cement 3D printing process experimentally. Through linear extrusion and rotary extrusion experiments, the main influencing factors and value ranges were determined. Experimental research showed that whether it is a straight path or a rotated path, the impact of extrusion/moving ratio on process quality is significant. Lombardi et al. [35] validated the ability to sense strand width in near real-time compared with in-line measurements, created a regression model of strand width versus print speed, and demonstrated closed-loop control of printed strand width using P and PI controllers, which were proved to improve printing quality, and this provides an idea for strand width in process control. Ertay and Altintas et al. [27] proposed a control strategy for comprehensive planning of tangential path velocity and material deposition rate. This control strategy synchronized the tangential velocity along the curved tool path in proportion to the material extrusion rate. Through the printing experiment of curved thermoplastic parts, it was proved that the synchronization of extrusion speed and moving speed can improve the printing quality. Jin et al. [26] proposed an optimization algorithm for equivalent parallel deposition path of MEX based on a horizontal set, and the local optimization strategy was adopted to solve the problem of uneven spacing between adjacent path segments. It was verified that the sedimentary path generated can effectively improve the sedimentary quality and obtain the variation trend of the contour by constructing the horizontal set function. Cerda-Avila et al. [9] compared multiple analytical models to predict the structural behavior of FDM parts. The results show it is feasible to use an analytical model to predict the structural behavior based on process parameters and material properties, which will reduce the need for existing experimental and numerical methods. Reference [21] optimized the process parameters of the FDM process using the gray Taguchi method and technique for order preference by similarity to ideal solution (TOPSIS) method. The effects of between layer height, infill method, build direction and processing time, surface roughness, and hardness was investigated, and multi-objective optimization of the above response parameters was achieved. On this basis, the strand spacing can be adjusted to uniform spacing, and the porosity can be reduced by local optimization. In particular, when printing rotated strands, the rotation radius also has a certain effect on the process quality. Experiments showed that this strand width control strategy can improve the quality of parts. The silicone MEX process can also be optimized by controlling the strand height. Layer height can be adjusted to improve surface quality, handling, peaks, and staircase effect [36], as well as features missing and other defects. 

However, there are few reports of methods considering both strand width and strand height, which can improve printing efficiency and accuracy of silicone MEX. In this study, the morphological model of strand cross-section was developed using moisture-cured silicone 3D printing, and a process optimization method considering strand morphology, denoted as SMO, which allows adjustment of the strand cross-section geometric shape by adjusting process parameters during the printing is proposed. This concept was applied to specimen manufacturing, and the effect of this method on product mechanical properties and accuracy was investigated through tensile tests and profile scans to verify its feasibility. 

## 2. Material and Methods

### 2.1. Material

In the present study, a commercially available one-component moisture-curing silicone elastomer (Dow Corning 737, Dow Corning, Midland, MI, USA) was utilized for all of the specimens. According to the datasheet, the silicone has a hardness of 33 Shore A, a tensile strength exceeding 1.2 MPa, and elongation exceeding up to 300%. The material has a zero-shear-rate viscosity of about 62.5 Pa·s. The silicone begins curing under exposure to atmospheric moisture. When it is exposed to this moisture, its skin coverage time, the tack-free time, and the cure to handing time are 3–6 min, 14 min, and 24 h, respectively [37].

### 2.2. AM Machine and Setup

**Figure 1:**
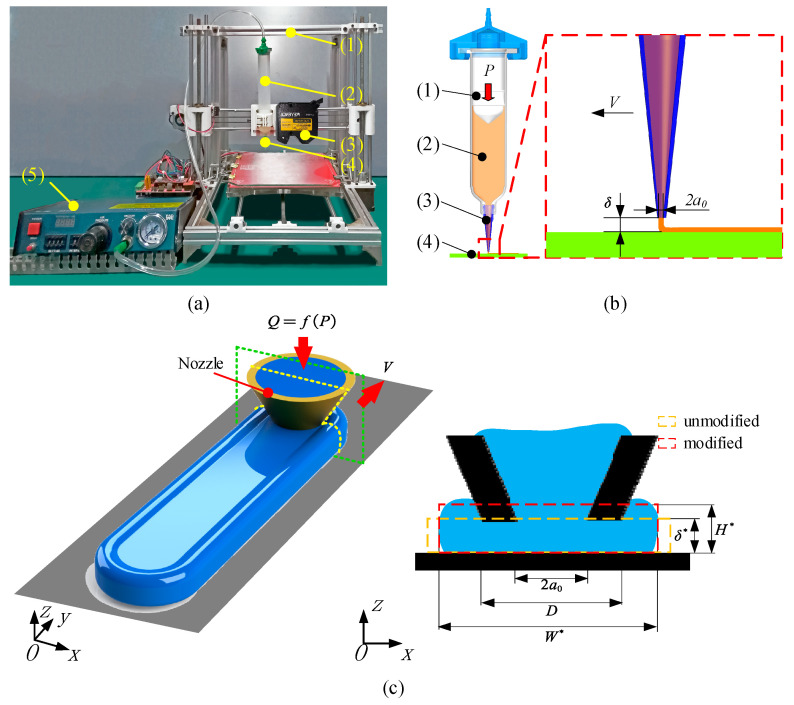
(**a**) AM machine: (1) motion control platform; (2) silicone container; (3) laser displacement sensor; (4) nozzle, which is used to replace (2) when scanning profiles; and (5) 982A fluid dispenser. (**b**) The schematic diagram of printing parameters: (1) piston; (2) silicone; (3) nozzle; (4) substrate, nozzle moving speed *V*, the inner diameter of nozzle tip 2*a*_0_ and gap between the nozzle tip and substrate δ. (**c**) Schematic diagram of strand width model correction.

The silicone AM system is shown in Figure 1a, which was used to print strands and fabricate tensile test specimens. The system consists of four key components, specifically (1) the motion control platform based on Reprap Prusa I3 (Nanjing puppet electronic technology Co. Ltd., Nanjing, Jiangsu, China); (2) 982A fluid dispensers (Dongmao company, Shanghai, China); (3) a tapered nozzle with 0.84 mm inner diameter; and (4) a silicone container, which is a transparent syringe barrel (Model Optimum by Nordson EFD, Westlake, OH, USA). The temperature during AM is between 20 and 25 °C and the humidity is between 50% and 60% to ensure that the cure rate is not artificially increased. Because silicone is a shear-thinning non-Newtonian fluid, it can maintain strand shape after extrusion, making it possible to manufacture layer by layer. Since the time to print one layer is shorter than the skin-over time (3–6 min) [37], strand to strand and layer to layer bonding can be achieved, and the probability of defects is greatly reduced. It should be indicated that the silicone is extruded directly through the nozzle onto the glass substrate.

### 2.3. Strand Width Model

A pressure-driven flow model was built to establish the relationship between air pressure and flow. Assuming that the extruded material is incompressible (not considering the depression of the cross-sectional morphology due to nozzle movement and hydrodynamics), the strand height between the extruded layers is layer height, and there should be no overlap or collapse between the extruded layers caused by gravity and hydrodynamic factors. Then, a strand width model was established, considering the nozzle height, moving speed, extrusion speed, and nozzle diameter, so that the relationship between process parameters and geometrical parameters of strand cross-section could be determined.

Firstly, regarding the liquid flow state at the nozzle, the theoretical flow rate is derived by establishing a pressure-driven flow model and setting the applied air pressure. The state of extruded fluid at the nozzle is limited by pressure, fluid gravity, surface tension, and friction [22]. If the balance of these forces is considered during steady flow, then a multivariable system of equations with infinite solutions is obtained. To simplify the model, assume that the driving force (air pressure P) is the dominant factor in the system, the other factors are ignored, the flow state is laminar flow, the fluid material is Bingham fluid, and the flow in the nozzle is given by the Poiseuille equation:

(1) $$Q=-\frac{\pi a_{0}^{4}}{8\mu }\frac{dp}{dz}$$

where Q is the flow rate of the fluid (silicone), $a_{0}$ is the inner radius of the nozzle tip, dp/dz is the applied pressure gradient, and μ is the viscosity of the fluid (silicone). Before the material is extruded at the nozzle, the critical pressure $P_{crit}$ needs to be reached, where the critical pressure is proportional to the viscosity of the liquid. When the shear stress is less than the critical shear stress, the fluid is in a stationary state and has a colloidal structure inside that can withstand the effect of stress; when the stress is greater than the critical value, the colloidal structure is destroyed, and the fluid begins to flow and be extruded out of the nozzle. In this model, the pressure gradient is approximately $dp/dz=\left(P_{crit}-\Delta P\right)/l_{z}$, where ΔP is the pressure difference across the nozzle and $l_{z}$ is the length of the conical part of the nozzle. Therefore, the formula (1) can be written as:

(2) $$Q=\frac{\pi a_{0}^{4}}{8\mu }\frac{\Delta P-P_{crit}}{l_{z}}$$

From another point of view, the extruded fluid is deposited as a strand with strand width W and strand height H, with a cross-sectional area of A, and different cross-sections appear when the extrusion rate and moving speed V change [28]. Thus, the following is obtained:

(3) $$Q=A\frac{dl}{dt}=AV$$

where the length of the deposited strand per unit time is dl/dt, and the moving speed of the nozzle relative to the bed is V. Suppose the average velocity of the fluid passing through the nozzle cross-section is U, so $U=Q/\pi a_{0}^{2}$, so

(4) $$\frac{U}{V}=\frac{A}{\pi a_{0}^{2}}$$

**Figure 2:**
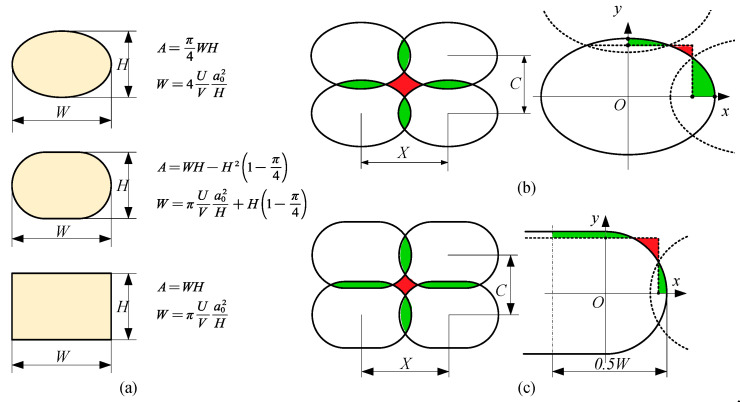
(**a**) Morphology of extruded strand cross-section, where W is strand width, H is strand height, V is moving speed of nozzle, U is the average velocity of the fluid passing through the nozzle cross-section, A is cross-sectional area of strand, $a_{0}$ is the inner radius of the nozzle tip, X is spacing of strands, and C is layer height. (**b**) No voids conditions for elliptical cross-section. (**c**) No voids conditions for rounded rectangle cross-section. The green part represents the overlap, and the red part represents the blank.

There are three assumptions for cross-sectional morphology, ellipse, rounded rectangle, and rectangle. Different assumptions correspond to different strand width expression. Through the expression of cross-sectional area and (3), the strand width model under different assumptions can be obtained as shown in Figure 2a.

Under certain conditions, the model can be used to predict the size of the strand width. The extrusion speed U, moving speed V, nozzle diameter $2a_{0}$, and nozzle height (theoretical layer height or the gap between nozzle tip and substrate) δ can be changed separately to adjust the width and height of the strand cross-section. Keeping the strand height δ=H in order to get a linear strand width model, assuming the cross-section is ellipses and rounded rectangles, $H<2\;a_{0}\;\sqrt{U/V}$ [28]; when assuming the cross-section is rectangular, $H<a_{0}\sqrt{\pi U/V}$.

After that, a linear strand width model is obtained, which can control the strand width by adjusting U, V, $a_{0}$, and δ. The strand spacing needs to be adjusted to make the overlapping part (green) fill the void part (red), so as to avoid voids as much as possible to maintain the mechanical properties. Assuming that the material is incompressible, and there is no overlap in the layer height direction, the strand spacing meets the constraint condition, and there are no voids between strands. The constraint condition is:

(5) $$X=\pi \frac{U}{V}\frac{a_{0}^{2}}{H}$$

### 2.4. Process Optimization Method Considering Strand Morphology (SMO)

Inspired by the adaptive slicing method, the concept of SMO is proposed. In the process of MEX, the process parameters can be controlled as required, and then the strand width or strand height can be changed to meet the requirements of non-uniform layer height or non-uniform strand spacing in the manufacturing process.

**Figure 3:**
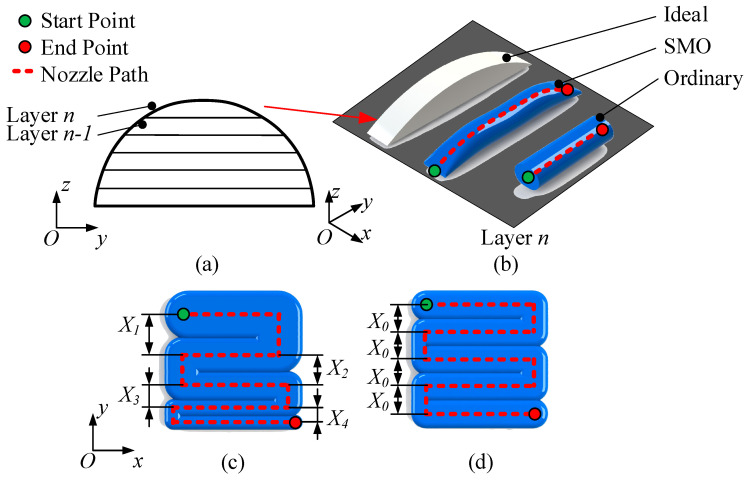
Schematic diagram of SMO method, where the green point is where the nozzle starts printing, the red point is where the nozzle ends printing, and the red dashed line is the nozzle trajectory. (**a**) Schematic diagram of example model, where the profile of the forming direction is a curve, and the model is divided into n layers. (**b**) Comparison of the ability to adjust strand height using different methods, where the product to fabricate is one layer in the example model, and SMO method can adjust the strand height within the range of the maximum and minimum strand height, thus avoiding staircase errors as much as possible, while the ordinary method only uses the set layer height, thus generating staircase errors. (**c**) Schematic diagram of the SMO strategy with strand width adjusted, where $X_{1}$, $X_{2}$, $X_{3}$, $X_{4}$ are the ununiform strand spacings. (**d**) Schematic diagram of the uniform strand width strategy, where $X_{0}$ is the uniformed strand spacing.

According to (5), U, V, and δ can be adjusted, so as to control W and H, to improve 3D printing quality. In this way, the staircase error can be reduced as much as possible, and the mechanical properties can be enhanced. For example, Figure 3 shows the adjustment of H and W. The green dots represent the printing start point, and corresponding red dots represent the printing stop point. Figure 3a,b show the adjustment of strand height (in the z-axis direction) using SMO. When printing a semi-circular part, layer n is as shown in Figure 3b. If using the ordinary slicing method with the layer height set as δ=H, the extruded strand is uniform with the same cross-sectional shape. If the SMO method is applied, H is adjusted within the range of $H_{\min}\sim \delta$, and the strand can be as close as possible to the target outline with elegant radian, as shown in Figure 3b. Obviously, with ordinary slicing, obvious staircase error occurs, but with SMO, staircase error can be reduced to a certain extent. In the XOY plane, as shown in Figure 3c,d, the adjustment of the strand width by the SMO method is shown. The basis of MEX is to use extruded strands to infill the pattern. When slicing, if utilizing the ordinary slicing method, only one strand spacing can be set, as shown in Figure 3d. When the part is under load or in other situations, the print quality is required to be improved, and the infilling should be adjusted accordingly. When SMO is utilized, the strand width and spacing can be adjusted to meet needs, as shown in Figure 3c.

## 3. Experimental Setup

The experiment was divided into two parts. Firstly, the strand width model was compared to determine the appropriate model for describing the strand cross-section, considering the extrusion process can be divided into steady-state and transient, and printing continuous strands is an effective method to obtain steady-state strand width [38]. After that, the concept of SMO was verified. Using the concept of SMO, specimens with strand width and height adjusted separately were designed and manufactured. Then, tensile tests and profile scans were conducted to further explore the impact of this method on mechanical properties and profile accuracy.

### 3.1. Continuous Strand Printing

**Figure 4:**
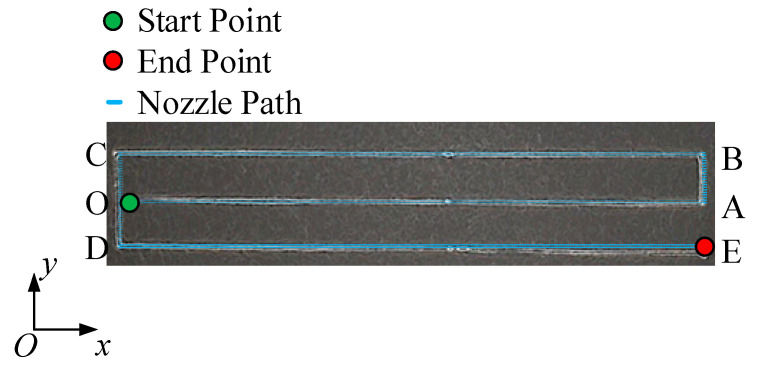
Schematic diagram of “E” pattern.

The trajectory was set to the shape of “e”, and the strand widths of BC and DE segments were measured to avoid the influence of printing direction on the results. The process parameters (as shown in Table 1) were set as follows: the backpressure P was adjusted to 172.369 kPa (25 Psi), V to 780~3420 mm/min with an interval of 660 mm/min, and δ to 0.2–1.0 mm with an interval of 0.2mm. After printing, the printed specimens were left completely exposed to air for 24 h to ensure they were fully cured, as shown in Figure 4. Steady-state strand width was then measured using a digital microscope and compared to predicted values. The focus of this study was to predict the parameters (strand width and strand height) that could be obtained by image processing during the printing process. This not only helps to improve printing performance and accuracy but also helps to achieve online control of strand width.

### 3.2. Strand Width Tunable Specimen Fabrication and Tensile Testing

Using the SMO concept, dumbbell specimens were designed and fabricated, and tensile tests were conducted to evaluate the feasibility of the concept.

Since the material we used was silicone rubber, dumbbell specimens were selected, and tensile tests were performed in accordance with the ISO 37:2017 standard. A 3D model of the silicone tensile test specimen was built using SolidWorks 2016 (Dassault Systèmes). Then, the 3D model was exported as a stereolithography (STL) file, and tool paths were generated with specific printing process parameters in G code. Then, the G code file was sent to the 3D printer to start printing. 

The dumbbell specimens were placed horizontally and infilled in 90 degrees vertically. Table 2 shows the printing parameters of dumbbell specimens. By adjusting the width X of the infill strand, different dumbbell samples can be obtained. In this research, four different types of specimens were designed. Three groups of them were uniformly filled with three different widths X. The width X was 0.8 mm (in 3.35 min), 1.0 mm (in 3.18 min) and 1.2 mm (in 3.58 min). Applying SMO, the fourth group specimen was divided into three segments. Each segment was evenly infilled with a different strand width X (in 3.22 min). The spacing of three parts of the strands was indicated by $X_{1}=1.2\;\mathrm{mm}$, $X_{2}=1.0\;\mathrm{mm}$, and $X_{3}=0.8\;\mathrm{mm}$ (from left to right). In addition, the length of the middle portion was 35 mm.

**Figure 5:**
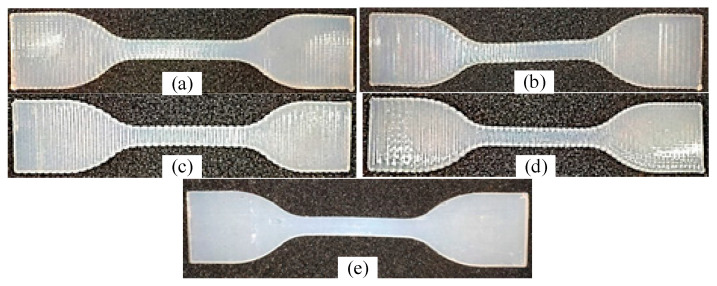
Detail of the AM specimens: (**a**) *X* = 0.8 mm, *F* = 2520 mm/min, *P* = 25 Psi; (**b**) *X* = 1.0 mm, *F* = 2100 mm/min, *P* = 25 Psi; (**c**) *X* = 1.2 mm, *F* = 1620 mm/min, *P* = 25 Psi; (**d**) SMO: *X*_1_ = 1.2 mm, *X*_2_ = 1.0 mm, *X*_3_ = 0.8 mm, *F*_1_ = 1620 mm/min, *F*_2_ = 2100 mm/min, *F*_3_ = 2520 mm/min, *P* = 25 Psi; (**e**) injection molded specimen. The spots are caused by dust.

**Figure 6:**
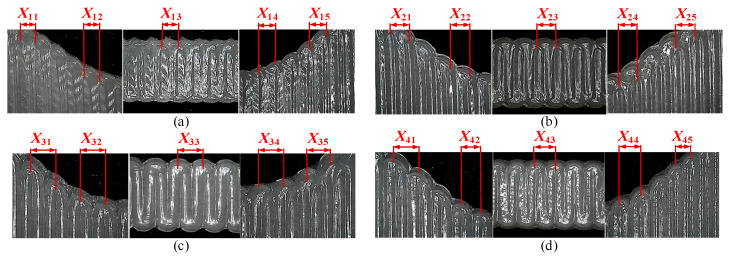
Partial enlarged detail of the AM specimens: (**a**) *X* = 0.8 mm, *F* = 2520 mm/min, *P* = 25 Psi; (**b**) *X* = 1.0 mm, *F* = 2100 mm/min, *P* = 25 Psi; (**c**) *X* = 1.2 mm, *F* = 1620 mm/min, *P* = 25 Psi; (**d**) SMO: *X*_1_ = 1.2 mm, *X*_2_ = 1.0 mm, *X*_3_ = 0.8 mm, *F*_1_ = 1620 mm/min, *F*_2_ = 2100 mm/min, *F*_3_ = 2520 mm/min, *P* = 25 Psi.

During the specimen fabricating process, the layer height C=0.5 mm, the inner diameter of the nozzle $2a_{0}=0.84\;\mathrm{mm}$, and the air pressure P=25 Psi. The layer height was selected mainly considering the need to divide the specimen thickness (2 mm) integrally to avoid features missing during slicing, which leads to the fabrication of specimens with a thickness less than the design value. Printing parameters are shown in Figure 1b. For basic tensile strength comparison, a group (three) of tensile test specimens (the same material as the AM specimen) were made by injecting silicone into a 3D printed baseline mold coated with mold release and taken out after curing. Their shape and thickness were similar to tensile test specimens fabricated by MEX. Figure 5 shows the specimens made for this research. Figure 6 shows the partial magnification details of each part of the specimens. It can be seen that $X_{11}=X_{12}=X_{13}=X_{14}=X_{15}=0.8\;\mathrm{mm}$; $X_{21}=X_{22}=X_{23}=X_{24}=X_{25}=1.0\;\mathrm{mm}$; $X_{31}=X_{32}=X_{33}=X_{34}=X_{25}=1.2\;\mathrm{mm}$; $X_{41}=1.2\mathrm{mm};\;X_{42}=X_{43}=X_{44}=1.0\;\mathrm{mm}$; and $X_{45}=0.8\;\mathrm{mm}$. Three identical specimens were made for each group to avoid the effect of randomness error.

**Figure 7:**
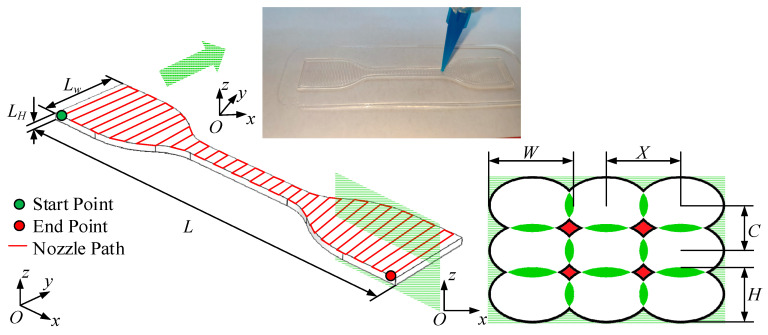
Silicone dumbbell specimen size and cross-sectional fill strand size. *X*, *C*, *W*, and *H* represent the fill strand spacing, the layer height, the strand width, and the nozzle height in the first layer, respectively. *L* = 115 mm, *L_W_* = 25 mm, *L_H_* = 2 mm.

A WDV-5A tensile testing machine (Wuxi Dumont Instrument Manufacturing Co., Ltd.) was used to conduct tensile test, under environmental conditions of 20–25 °C, 20–60% humidity, and a speed of 10 mm/min, according to ISO 37:2017. Different printing directions affect the strength of the product when printing sheet-like parts [39]. If the sheet-like specimen is placed upright, the printing process tends to be unstable as the height increases, which finally causes printing failure. In order to provide better mechanical properties, the specimen was placed horizontally (XOY plane), as shown in Figure 7. In order to study whether SMO sacrifices the tensile strength of specimens, the dumbbell specimen parametrically designed according to SMO was fabricated and compared with uniform infill, injection-molded ones. In addition, this experiment focused on the effect of uneven strand spacing (strand width was determined according to the strand width model and no void constraints) on the tensile strength of the specimen. Figure 7 shows the dimensions of the silicone specimens.

### 3.3. Strand Height Tunable Specimen Fabrication and Profile Scanning

**Figure 8:**
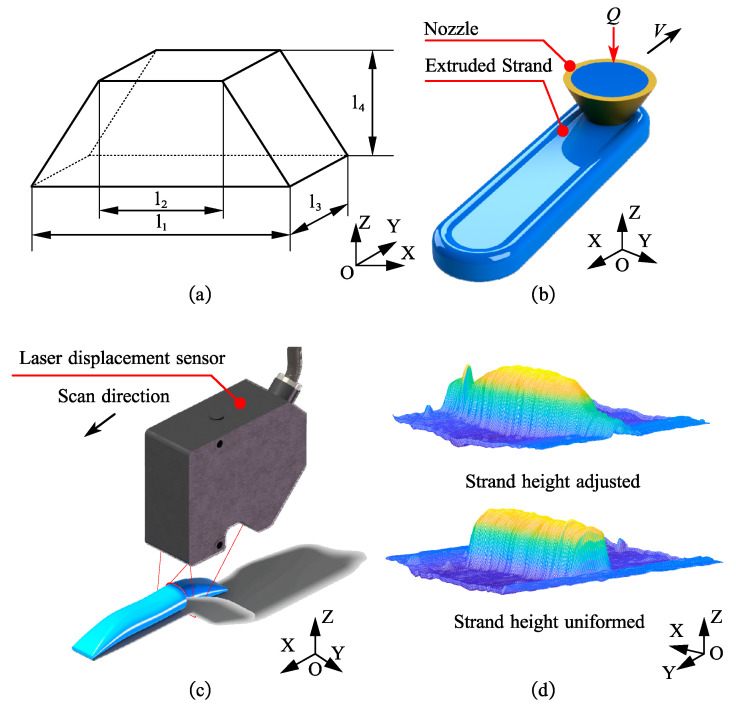
Schematic diagram of specimen fabrication and profile scanning with adjustable strand height. (**a**) 3D model of the specimen, where l1 = 60 mm, l2 = 20 mm, l3 = 1.0 mm, l4 = 0.8 mm; (**b**) schematic diagrams of the specimen fabrication process; (**c**) profile scanning process, where the data of each profile scan consist of 800 height measurements distributed along the Y-axis, and the laser displacement sensor moves one small step along the scanning direction after each profile scan is completed, until the specimen is scanned; (**d**) strand profiles obtained by scanning specimens printed by different methods, where the 3D model of the manufactured specimen is obtained by data processing of the contour data.

The specimen was designed as shown in Figure 8a, and its main view is an isosceles trapezoid. As in many designs, the specimen is set such that the height is a non-integer multiple of the set strand height (0.6 mm), and there is a continuous variation in the side profile to verify the feasibility of the proposed method. The process parameters were set as 2*a*_0_ = 0.84 mm, *V* = 46 mm/s, δ = 0.6 mm, *P* = 25 Psi, followed by the generation of G-codes using the uniform layer height and SMO methods. The former does not modify the initial parameters, while the latter controls the minimum strand height of 0.2 mm and the maximum strand height of 0.84 mm and adjusts the moving speed according to the model established in the first experiment in order to maintain the strand width when adjusting the strand height during the printing process. Extrusion was started earlier and stopped later in the experiment to avoid a time delay effect in the silicone MEX process, and this part is ignored in the data processing. After the printing was completed, the profile data of the specimen were collected using a Keyence LJ-G015 laser displacement sensor, and the distribution of the strand height with the printing direction was obtained after data processing. Its resolution is 0.2 μm in the Z-direction and 2.5 μm in the Y-direction. Then, the strand height contour error, denoted as strand height error, was defined as the maximum error between the experimental value of strand height and the theoretical curve and calculated. The above process was repeated three times to avoid the effect of randomness error.

## 4. Results and Discussion

### 4.1. Strand Width Model

**Figure 9:**
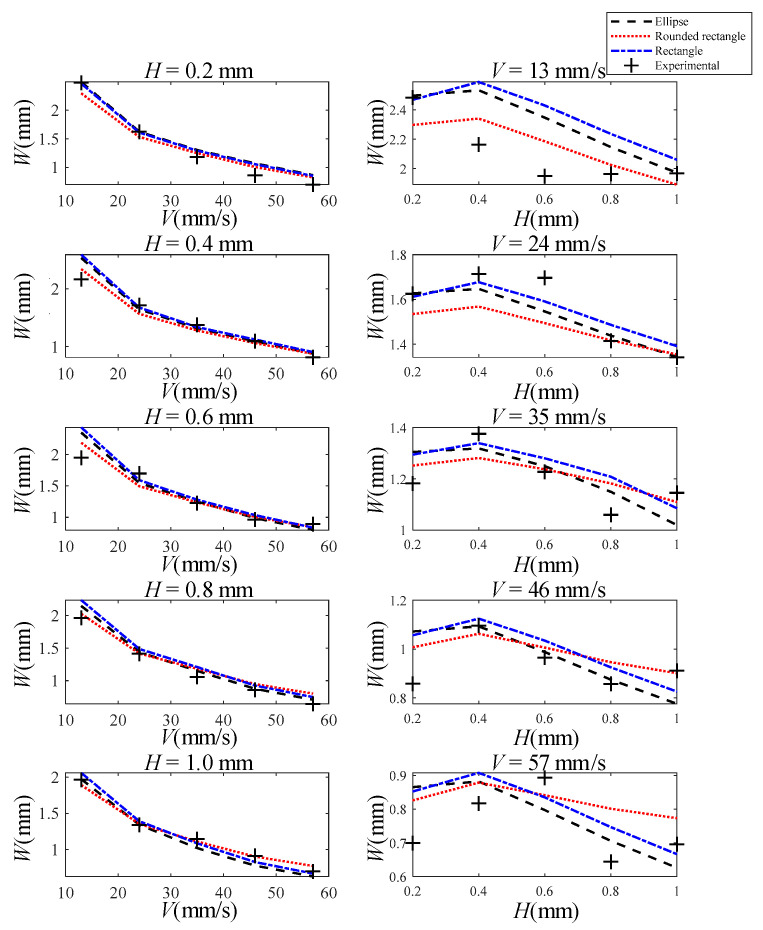
Experimental and theoretical results of strand width with different process parameters, where the black dashed line is the predicted strand width assuming an elliptical cross-section shape, the red dotted line is the predicted strand width assuming a rounded rectangular cross-section shape, the blue dash-dotted line is the predicted strand width assuming a rectangular cross-section shape, and the black plus sign is the experimentally measured value. The graphs in the first column show the relationship between strand width and moving speed of nozzle for different nozzle heights (0.2, 0.4, 0.6, 0.8, 1.0 mm); The second column of graphs shows the relationship between strand width and nozzle height for different moving speeds (13, 24, 35, 46, 57 mm/s).

Strand width data under different process parameters were measured after experiments, as shown in Figure 9. Considering the actual value of the nozzle height (equal to strand height) includes the set value and error $\delta^{*}=\delta +k_{b}$, and the extrusion is driven by pressure, when the nozzle height is small, head loss occurs, resulting in the actual extrusion speed being a function of the actual nozzle height. The corrected extrusion rate is standardized to $U^{*}=U\times \left(1/\left(1+d/\delta^{*}\right)k_{a}+k_{c}\right)$. In addition, the strand width (red dotted frame) is smaller than the theoretical value (orange dotted frame) when there is too much material accumulation ($H^{*}>\delta^{*}$), as shown in Figure 1c. Thus, when the theoretical strand width W is larger than the outer diameter of the nozzle D, the empirical formula $W^{*}=a_{0}+0.5W$ is used to correct for it. 

Comparing the theoretical solution with the experimental values, it is found that $R^{2}=0.94071$ when the cross-section is assumed to be elliptical, $R^{2}=0.95127$ when the cross-section is assumed to be a rounded rectangle, and $R^{2}=0.94058$ when the cross-section is assumed to be rectangular. The results show that no matter what cross-sectional morphology assumption is used, each model can fit the true strand width well. Among them, the rounded rectangular cross-section model is the best model for strand width, as its $R^{2}$ value is the largest.

### 4.2. Strand Width Tunable Specimen Fabrication and Tensile Testing

**Figure 10:**
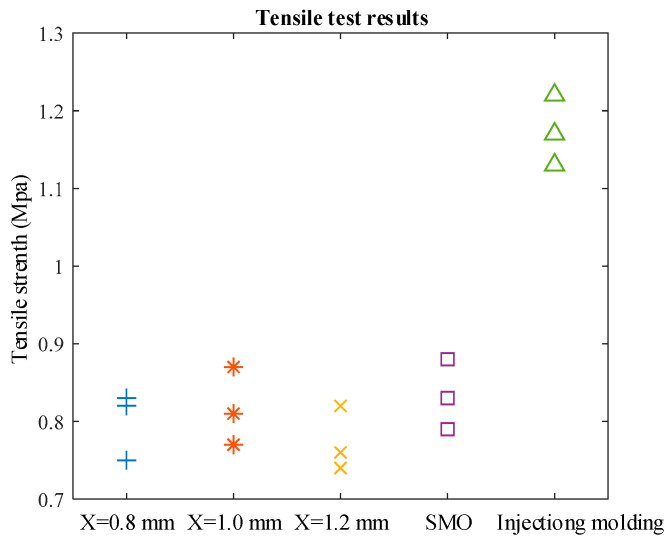
Results of tensile tests, where the blue plus sign shows the tensile strength of the specimen with a strand spacing of 0.8 mm, the red asterisk shows the tensile strength of the specimen with a strand spacing of 1.0 mm, the yellow cross shows the tensile strength of the specimen with a strand spacing of 1.2 mm, the purple square shows the tensile strength of the specimen made by the SMO method, and the green upward-pointing triangle shows the tensile strength of the specimen made by injection molding.

Tensile tests were carried out on specimens, and different tensile strength values were obtained and recorded in Figure 10 and Table 3. 

Five groups of tensile tests were conducted, and three specimens were selected in each group to avoid random errors. It is found that the difference in tensile strength of 3D printed specimens is not significant, ranging from 0.75 to 0.85 MPa. In addition, the specimens applying SMO perform well among the above specimens. The results show that the lowest tensile strength (average: 0.77 MPa) was obtained with a strand width of 1.2 mm, a better tensile strength was obtained with a stand width of 1.0mm (average: 0.81 MPa) and the SMO method (average: 0.83 MPa), and the tensile strength of the injection-molded specimen as a control was significantly higher than that of the sample through the additive manufacturing process (average: 1.17 MPa).

In order to compare the efficiency of printing methods, the ratio of tensile strength to printing time was used as the evaluation coefficient. The larger the ratio, the higher the efficiency. Table 3 gives the evaluation results. The results show that there is no significant difference in efficiency between the SMO method and the common method. When the strand spacing is 1.0 mm and the SMO is used, higher coefficients (0.252 and 0.258) can be obtained. Considering that injection molding requires 24 h of curing, the mold needs to be printed in advance, and the time of related operations, which makes it difficult to calculate the printing time, there is no specific value for the coefficients. However, it is obvious that the time taken for injection molding is measured in hours, and although the highest tensile strength is obtained, its efficiency is the lowest.

### 4.3. Strand Height Tunable Specimen Fabrication and Profile Scanning

**Figure 11:**
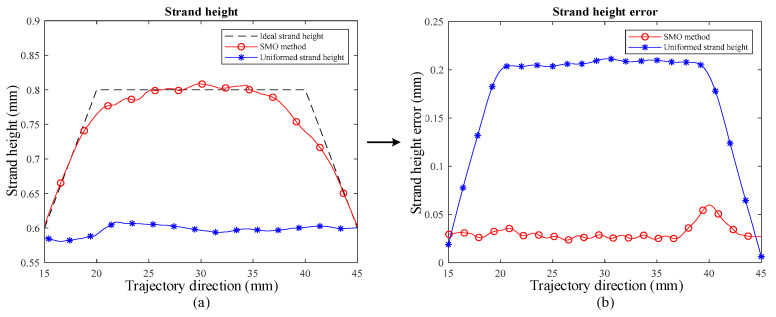
Strand height and strand height error distribution. (**a**) Strand height distribution along the trajectory, where the red curve with circles is the distribution of strand height along the trajectory direction for specimens made using the SMO method, and the blue curve with asterisks is the distribution of strand height along the trajectory direction for specimens made using the uniform strand height; (**b**) strand height error distribution along the trajectory, where the red curve with circles is the distribution of strand height error along the trajectory direction for specimens made using the SMO method, and the blue curve with asterisks is the distribution of strand height error along the trajectory direction for specimens made using the uniform strand height method.

The three groups of specimens were scanned by the laser displacement sensor at 0.1 mm intervals in the scan direction, with anomalies removed, and the strand surface morphology data were obtained, as shown in Figure 8d. Then, the strand section contour was extracted along the trajectory direction, the maximum value of the contour was used as the strand height, and the distribution of the strand height along the trajectory of the specimens printed using different methods was obtained, as shown in Figure 11a. The distribution of the strand height error along the trajectory of the specimens printed using different methods was calculated according to the definition, as shown in Figure 11b, along with its maximum value, mean value, and standard deviation to evaluate the printing accuracy.

Among them, the maximum value of strand height error for the specimen printed using uniform strand height is 0.2133 mm, the mean value is 0.1728 mm, and the standard deviation is 0.1532 mm. The maximum value of strand height error for the specimen printed using the SMO method is 0.0737 mm, the mean value is 0.0310 mm, and the standard deviation is 0.0151 mm. It is observed that the mean value of strand height error and the mean square deviation for the SMO method are much smaller than the other method, which indicates that the overall error of the SMO method is smaller and more uniformly distributed, which can also be observed in the figure. The above results indicate that the SMO method with the strand height adjusted provides higher strand accuracy compared to the uniform strand height.

Furthermore, it is observed that in Figure 11a, the corners of the ideal strand height have a larger error than the strand height corresponding to the SMO method, which is also shown in Figure 11b. This is thought to be due to the material properties of the silicone material, such as time delay and tension, so avoiding abrupt changes in the contour strand in the direction of the layer height at the design stage will help to improve the contour accuracy of the product, which is a guideline for reducing the strand height error.

## 5. Conclusions

The strand width model of MEX was studied, and the model could fit the experimental data well, indicating the model could be used. In addition, the differences caused by different cross-section assumptions (ellipse, rounded rectangle, rectangle) were not significant. When using rounded rectangles, the model fits best. This model will provide a theoretical basis for the control of strand width and strand height.

Based on the strand width model, the concept of SMO is proposed, and two application scenarios are described. To initially verify this concept, two experiments were designed with SMO, adjusting the strand width and strand height, to investigate the effect of SMO on the tensile strength of the printed product, as well as the strand accuracy. The results showed that the SMO method with adjustable strand width can effectively balance efficiency and mechanical properties compared to uniform infill, and the SMO method with the strand height adjusted provides higher strand accuracy compared to the uniform strand height strategy. This verifies the possibility of SMO and illustrates the feasibility of the method to a certain extent. According to the perspective of the production cycle, cost, operability, and geometry range of silicone products, silicone 3D printing technology has significant advantages.

The results of this study can be used to guide the control of the strand width and strand height of MEX and are not limited to silicone materials, such as cement. However, when the nozzle moving speed is less than 24 mm/s or more than 46 mm/s, the proposed model cannot predict the strand cross-section shape well; the strand height error at the corners increases using the SMO method. Future research should expand the applicability of the strand cross-section model and investigate the mechanism of increasing error at the corners.

## Figures and Tables

**Table 1:** Continuous strand printing parameters.

	Nozzle Height δ	Moving Speed V	Constant Factors
Parameters	0.2~1.0 mm Interval: 0.2 mm	780~3420 mm/min Interval: 660 mm/min	P = 172.369 kPa $2a_{0}$ = 0.84 mm

**Table 2:** The printing parameters of the dumbbell specimens.

Layer Height (*C*)	Pressure (*P*)	Feed Speed (*F*)	Strand Width (*X*)
**0.5 mm**	25 Psi	2520 mm/min	0.8 mm
**0.5 mm**	25 Psi	2100 mm/min	1.0 mm
**0.5 mm**	25 Psi	1620 mm/min	1.2 mm
**0.5 mm**	25 Psi	1620~2520 mm/min	0.8~1.2 mm

**Table 3:** Printing efficiency of different filling methods.

Dumbbell Specimen	Tensile Strength σ	Printing Time $t_{p}$	Ratio $i_{\sigma t}=\sigma /t_{p}$
**Filled Uniformly, *X* = 0.8 mm**	0.8 MPa	3.35 min	0.233
**Filled Uniformly, *X* = 1.0 mm**	0.81 MPa	3.18 min	0.252
**Filled Uniformly, *X* = 1.2 mm**	0.77 MPa	3.58 min	0.212
**SMO Method**	0.83 MPa	3.22 min	0.258
**Injection Molding**	1.17 MPa	/	/

## Data Availability

The data that support the findings of this study are available from the corresponding authors upon reasonable request.

## Nomenclature

U	The average extrusion velocity inside the nozzle
V	Moving speed of nozzle relative to substrate
δ	The gap between nozzle tip and substrate
$a_{0}$	Inner radius of the nozzle tip
W	Strand width
H	Strand height
A	Cross-sectional area of strand
X	Spacing of strand
C	Layer height
P	Air pressure
Q	Flow rate

## References

[1] Liravi F., Toyserkani E. (2018). Additive manufacturing of silicone structures: A review and prospective. Addit. Manuf..

[2] Kolesky D.B., Truby R.L., Gladman A.S., Busbee T.A., Homan K.A., Lewis J.A. (2014). 3D bioprinting of vascularized, heterogeneous cell-laden tissue constructs. Adv. Mater. Weinheim..

[3] Zhou L.-Y., Gao Q., Fu J.-Z., Chen Q.-Y., Zhu J.-P., Sun Y., He Y. (2019). Multimaterial 3D Printing of Highly Stretchable Silicone Elastomers. ACS Appl. Mater. Interfaces.

[4] Plott J., Tian X., Shih A.J. (2018). Voids and tensile properties in extrusion-based additive manufacturing of moisture-cured silicone elastomer. Addit. Manuf..

[5] Li D.-R., Tian X., Wang H., Plott J., Shih A. (2018). Five-Axis Extrusion-Based Additive Manufacturing of Silicone 3D Contour Nonwoven Fabrics. Proceedings of the 13th International Manufacturing Science and Engineering Conference.

[6] Khorasani M., Ghasemi A., Rolfe B., Gibson I. (2021). Additive manufacturing a powerful tool for the aerospace industry. Rapid Prototyp. J..

[7] Wang X., Xu S., Zhou S., Xu W., Leary M., Choong P., Qian M., Brandt M., Xie Y.M. (2016). Topological design and additive manufacturing of porous metals for bone scaffolds and orthopaedic implants: A review. Biomaterials.

[8] Messimer S., Pereira T., Patterson A., Lubna M., Drozda F. (2019). Full-Density Fused Deposition Modeling Dimensional Error as a Function of Raster Angle and Build Orientation: Large Dataset for Eleven Materials. J. Manuf. Mater. Process..

[9] Cerda-Avila S.N., Medellín-Castillo H.I., Lim T. (2021). Analytical models to estimate the structural behaviour of fused deposition modelling components. RPJ.

[10] Yang S., Tang Y., Zhao Y.F. (2015). A new part consolidation method to embrace the design freedom of additive manufacturing. J. Manuf. Process..

[11] Duoss E.B., Weisgraber T.H., Hearon K., Zhu C., Small W., Metz T.R., Vericella J.J., Barth H.D., Kuntz J.D., Maxwell R.S. (2014). Three-Dimensional Printing of Elastomeric, Cellular Architectures with Negative Stiffness. Adv. Funct. Mater..

[12] Foerster A., Wildman R., Hague R., Tuck C. Reactive inkjet printing approach towards 3D silcione elastomeric structures fabrication. Proceedings of the 28th Annual International Solid Freeform Fabrication Symposium.

[13] Wu Y., Yang J., Zhu X., Tang C., Liu T., Liu Y., Mei J. (2018). Preparation of ZnO whisker reinforced silicone foam by 3D printing. Silicone Mater..

[14] Zhu X. (2019). Mechanical Behavior of Silicone Foams with Woodpile Structure Printed by Direct Ink Writing Technology. Ph.D. Thesis.

[15] Wacker Chemie A.G. ACEO® Introduces 3D Printing With Electrically Conductive Silicone Rubber. https://www.aceo3d.com/aceo-introduces-3d-printing-with-electrically-conductive-silicone-rubber/.

[16] Plott J., Shih A. (2017). The extrusion-based additive manufacturing of moisture-cured silicone elastomer with minimal void for pneumatic actuators. Addit. Manuf..

[17] Tian X., Plott J., Wang H., Zhu B., Shih A.J., Shih A., Cao J. (2017). Silicone Foam Additive Manufacturing by Liquid Rope Coiling. Proceedings of the 3rd CIRP Conference on Biomanufacturing, Chicago, IL, USA, 11–14 July 2017.

[18] Yuk H., Zhao X. (2018). A New 3D Printing Strategy by Harnessing Deformation, Instability, and Fracture of Viscoelastic Inks. Adv. Mater. Weinheim..

[19] Lalegani Dezaki M., Mohd Ariffin M.K.A., Hatami S. (2021). An overview of fused deposition modelling (FDM): Research, development and process optimisation. Rapid Prototyp. J..

[20] Bakır A.A., Atik R., Özerinç S. (2021). Mechanical properties of thermoplastic parts produced by fused deposition modeling: A review. Rapid Prototyp. J..

[21] Sakthivel Murugan R., Vinodh S. (2021). Parametric optimization of fused deposition modelling process using Grey based Taguchi and TOPSIS methods for an automotive component. Rapid Prototyp. J..

[22] Vozzi G., Previti A., de Rossi D., Ahluwalia A. (2002). Microsyringe-Based Deposition of Two-Dimensional and Three-Dimensional Polymer Scaffolds with a Well-Defined Geometry for Application to Tissue Engineering. Tissue Eng..

[23] Luis E., Pan H.M., Sing S.L., Bastola A.K., Goh G.D., Goh G.L., Tan H.K.J., Bajpai R., Song J., Yeong W.Y. (2019). Silicone 3D Printing: Process Optimization, Product Biocompatibility, and Reliability of Silicone Meniscus Implants. 3D Print. Addit. Manuf..

[24] Plott J., Tian X., Shih A. (2018). Measurement and Modeling of Forces in Extrusion-Based Additive Manufacturing of Flexible Silicone Elastomer With Thin Wall Structures. J. Manuf. Sci. Eng.-Trans. ASME.

[25] Wasserfall F., Hendrich N., Zhang J. Adaptive Slicing for the FDM Process. Proceedings of the 13th IEEE International Conference on Automation Science and Engineering (CASE).

[26] Jin Y., Du J., Ma Z., Liu A., He Y. (2017). An optimization approach for path planning of high-quality and uniform additive manufacturing. Int. J. Adv. Manuf. Technol..

[27] Ertay D.S., Yuen A., Altintas Y. (2018). Synchronized material deposition rate control with path velocity on fused filament fabrication machines. Addit. Manuf..

[28] Comminal R., Serdeczny M.P., Pedersen D.B., Spangenberg J. (2018). Numerical modeling of the strand deposition flow in extrusion-based additive manufacturing. Addit. Manuf..

[29] Comminal R., Serdeczny M.P., Pedersen D.B., Spangenberg J. (2019). Motion planning and numerical simulation of material deposition at corners in extrusion additive manufacturing. Addit. Manuf..

[30] Liravi F., Toyserkani E. (2018). A hybrid additive manufacturing method for the fabrication of silicone bio-structures: 3D printing optimization and surface characterization. Mater. Des..

[31] Muthusamy M., Safaee S., Chen R. (2018). Additive Manufacturing of Overhang Structures Using Moisture-Cured Silicone with Support Material. J. Manuf. Mater. Process..

[32] Miao J., Li F., He X., Xiang S., Ma H., Jiao Z., Yang W. (2019). Analysis of Molding Quality of 3D Printing Metal Powder/PVA Composite Slurry. Plastic.

[33] Chi B., Ma H., Liu X., Wang C., Jiao Z., Yang W. (2017). The Influence of 3D Printing Process Parameters on the Mechanical Behavior of TPU Products. Plastic.

[34] Foteinopoulos P., Esnault V., Komineas G., Papacharalampopoulos A., Stavropoulos P. (2020). Cement-based additive manufacturing: Experimental investigation of process quality. Int. J. Adv. Manuf. Technol..

[35] Lombardi J.P., Salary R., Weerawarne D.L., Rao P.K., Poliks M.D. (2019). Image-Based Closed-Loop Control of Aerosol Jet Printing Using Classical Control Methods. J. Manuf. Sci. Eng.-Trans. ASME.

[36] Zhou M.Y., Xi J.T., Yan J.Q. (2004). Adaptive direct slicing with non-uniform cusp heights for rapid prototyping. Int. J. Adv. Manuf. Technol..

[37] DOWSIL™ 737 Neutral Cure Sealant. https://www.dow.com/content/dam/dcc/documents/en-us/productdatasheet/95/95-10/95-1073-dowsil-737-neutral-cure-sealant.pdf?iframe=true.

[38] Li W., Ghazanfari A., Leu M.C., Landers R.G. (2017). Extrusion-on-demand methods for high solids loading ceramic paste in freeform extrusion fabrication. Virtual Phys. Prototyp..

[39] Abbott A.C., Tandon G.P., Bradford R.L., Koerner H., Baur J.W. (2018). Process-structure-property effects on ABS bond strength in fused filament fabrication. Addit. Manuf..

